# The Effects of Melittin and Apamin on Airborne Fungi-Induced Chemical Mediator and Extracellular Matrix Production from Nasal Polyp Fibroblasts

**DOI:** 10.3390/toxins9110348

**Published:** 2017-10-27

**Authors:** Seung-Heon Shin, Mi-Kyung Ye, Sung-Yong Choi, Kwan-Kyu Park

**Affiliations:** 1 Department of Otolaryngology-Head and Neck Surgery, School of Medicine, Catholic University of Daegu, Daegu 42472, Korea; miky@cu.ac.kr (M.-K.Y.); iroom-ent@naver.com (S.-Y.C.); 2 Department of Pathology, School of Medicine, Catholic University of Daegu, Daegu 42472, Korea; kkpark@cu.ac.kr

**Keywords:** melittin, apamin, *Alternaria*, *Aspergillus*, nasal fibroblast, chemical mediator, extracellular matrix

## Abstract

Melittin and apamin are the main components of bee venom and they have been known to have anti-inflammatory and anti-fibrotic properties. The aim of this study was to evaluate the effect of melittin and apamin on airborne fungi-induced chemical mediator and extracellular matrix (ECM) production in nasal fibroblasts. Primary nasal fibroblasts were isolated from nasal polyps, which were collected during endoscopic sinus surgery. Nasal fibroblasts were treated with *Alternaria* and *Aspergillus*. The effects of melittin and apamin on the production of interleukin (IL)-6 and IL-8 were determined with enzyme linked immunosorbent assay. ECM mRNA and protein expressions were determined with the use of quantitative RT-PCR and Western blot. *Alternaria*-induced IL-6 and IL-8 production was significantly inhibited by apamin. However, melittin did not influence the production of IL-6 and IL-8 from nasal fibroblasts. Melittin or apamin significantly inhibited collagen type I, TIMP-1, and MMP-9 mRNA expression and protein production from nasal fibroblasts. Melittin and apamin inhibited *Alternaria*-induced phosphorylation of Smad 2/3 and p38 MAPK. Melittin and apamin can inhibit the fungi-induced production of chemical mediators and ECM from nasal fibroblasts. These results suggest the possible role of melittin and apamin in the treatment of fungi induced airway inflammatory diseases.

## 1. Introduction

Nasal polyps are swellings and there is damage to the mucosal epithelium with inflammatory cell infiltration. Although the pathogenesis of nasal polyps is not fully understood, mucosal epithelial damage, extracellular matrix (ECM) accumulation, and increased local inflammatory mediators are the characteristic pathophysiologic findings of nasal polyps [1]. Fibroblasts are the main structural components of the nasal mucosa and they participate in the local immune response through the production of biological mediators which are involved in the recruitment of inflammatory cells and the cellular source of ECM proteins [2].

Fungi are ubiquitous saprophytes in nature and airway mucosa is exposed by inhalation of fungal spores. A fine immunologic balance to maintain a stable host–fungi relationship is important in order to maintain the physiologic condition, and disruption of the host immune response causes pathologic conditions of the airway. Fungi are commonly found in the nasal mucosa and relatively few species are implicated in airway inflammatory disease. *Alternaria* and *Aspergillus* are known common pathogens found in nasal secretion and they induce the production of chemical mediators from nasal epithelial cells and fibroblasts [3,4]. TLR2, TLR4, and TLR5 seem to be important pattern recognition receptors for fungi [3,5].

Bee venom (BV) has been used to treat several inflammatory diseases, such as rheumatoid arthritis, tendonitis, and Parkinson’s disease [6,7]. BV is a complex mixture with peptides, enzymes, and biogenic amines with various pharmaceutical properties. Melittin and apamin are the main components of BV peptide. Melittin comprises about 40–50% of the dried BV and has antibacterial, antiviral, anti-inflammatory, and anti-fibrotic properties [8,9,10]. Apamin also has anti-inflammatory and anti-fibrotic properties in various cells [11].

Melittin and apamin have anti-fibrotic activities that suppress the pro-fibrotic gene and protein expression through the inhibition of TGF-βRII-Smad, ERK1/2, and JNK phosphorylation in rat kidney fibroblasts [10]. Melittin and apamin have immunomodulatory activities, and in this study, we evaluated the effect of melittin and apamin on airborne fungi induced chemical mediator and ECM production in nasal fibroblasts.

## 2. Results

### 2.1. The Cytotoxicity of BV, Melittin, and Apamin

**Figure 1:**
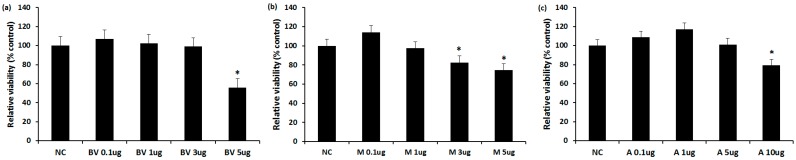
Effect of bee venom, melittin, and apamin on the proliferation of nasal fbroblasts. Nasal fibroblasts were treated with various concentrations of various concentrations of (**a**) bee venom; (**b**) melittina; and (**c**) apamin for 24 h. Values are expressed as the mean ± SE of four independent experiments. 5 μg/mL of BV, 3 μg/mL of melittin, and 10 μg/mL of apamin inhibited the proliferation of nasal fibroblasts. NC: negative control; BV: bee venom; M: melittin; A: apamin; μg: μg/mL; * *p* < 0.05.

MTT assay was used to determine the cytotoxicity of these three agents. The cells were treated with various concentrations of BV (0.1 to 5 μg/mL), melittin (0.1 to 5 μg/mL), and apamin (0.1 to 10 μg/mL) for 24 h. The viability of fibroblasts was significantly suppressed by BV at a concentration of 5 μg/mL (55.8 ± 6.8%), melittin at a concentration of 3 μg/mL (82.6 ± 8.3%), and apamin at a concentration of 10 μg/mL (55.8 ± 11.7%) (Figure 1). Based on these results, we used up to 3 μg/mL of BV, 1 μg/mL of melittin, and 5 μg/mL of apamin for further experiments. 

### 2.2. The Effect of BV, Melittin, and Apamin on the Production of Chemical Mediators

**Figure 2:**
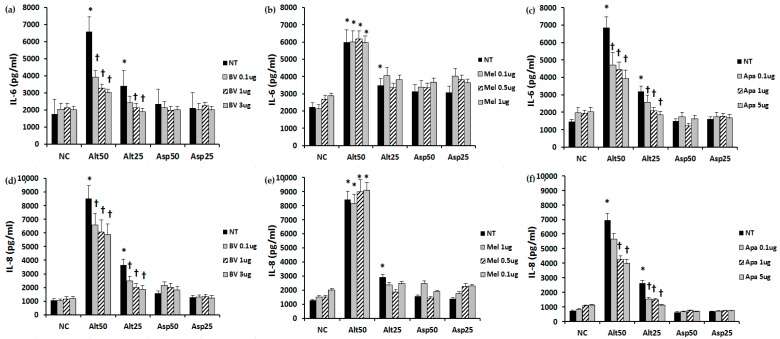
Effect of bee venom, melittin, and apamin on the production of IL-6 and IL-8 from nasal fibroblasts. Nasal fibroblasts were treated with *Alternaria* and *Aspergillus* for 24 h with or without various concentrations of these three agents. (**a**,**d**) *Alternaria* enhanced IL-6 and IL-8 production from nasal fibroblasts and the production of IL-6 and IL-8 was significantly inhibited by bee venom and (**c**,**f**) apamin; (**b**,**e**) Melittin did not influence the production of IL-6 and IL-8 from nasal fibroblasts. Alt 50: *Alternaria* 50 μg/mL; Asp 50: *Aspergillus* 50 μg/mL; NC: negative control; NT: non-treated; BV: bee venom; Mel: melittin; Apa: apamin, μg: μg/mL; * *p* < 0.05 compared with negative control; † *p* < 0.05 compared with the non-treated group.

When the fibroblasts were stimulated with *Alternaria*, IL-6 (6476.1 ± 352.4 pg/mL at a concentration of 50 μg/mL, 3368.1 ± 247.5 pg/mL at a concentration of 25 μg/mL) and IL-8 (7969.4 ± 690.2 pg/mL at a concentration of 50 μg/mL, 2399.0 ± 175.5 pg/mL at a concentration of 25 μg/mL) production was significantly increased compared with that in the non-stimulated group (IL-6; 1812.1 ± 93.6 pg/mL, IL-8; 1010.6 ± 132.4 pg/mL). However, *Aspergillus* did not significantly enhance the production of IL-6 and IL-8 from nasal fibroblasts. IL-6 and IL-8 production induced by *Alternaria* was significantly inhibited by BV and apamin in a dose dependent manner. However, melittin did not influence the production of IL-6 and IL-8 from nasal fibroblasts (Figure 2).

### 2.3. The Effect of Melittin and Apamin on the Expression of ECM 

**Figure 3:**
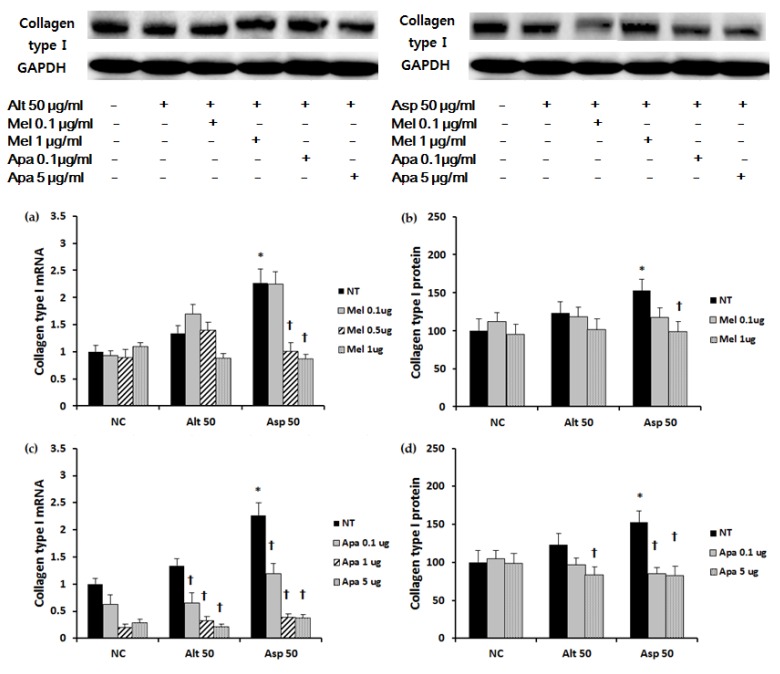
Effect of melittin and apamin on the expression of collagen type I mRNA and protein in nasal fibroblasts. *Aspergillus* induced collagen type I mRNA and protein expressions were significantly suppressed by (**a**,**b**) melittin and (**c**,**d**) apamin. Apamin also significantly suppressed collagen type I mRNA expression in negative control and the *Alternaria* stimulated group. Alt 50: *Alternaria* 50 μg/mL; Asp 50: *Aspergillus* 50 μg/mL; NC: negative control; NT: non-treated; Mel: melittin; Apa: apamin; μg: μg/mL; *: *p* < 0.05 compared with negative control; † *p* < 0.05 compared with the non-treated group.

**Figure 4:**
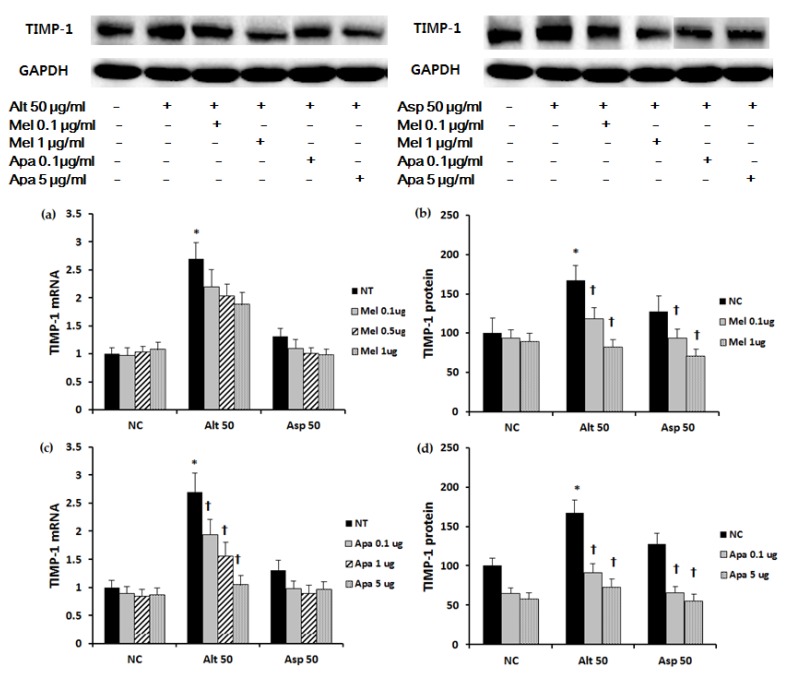
Effect of melittin and apamin on the expression of TIMP-1 mRNA and protein in nasal fibroblasts. *Alternaria* induced TIMP-1 protein production was significantly inhibited by (**b**) melittin and (**d**) apamin; (**c**) Apamin also significantly suppressed *Alternaria* induced TIMP-1 mRNA expression; (**a**) Melittin did no inhibit *Alternaria* induced TIMP-1 mRNA expression. Alt 50: *Alternaria* 50 μg/mL; Asp 50: *Aspergillus* 50 μg/mL; NC: negative control; NT: non-treated; Mel: melittin; Apa: apamin, μg: μg/mL; * *p* < 0.05 compared with negative control; † *p* < 0.05 compared with the non-treated group.

**Figure 5:**
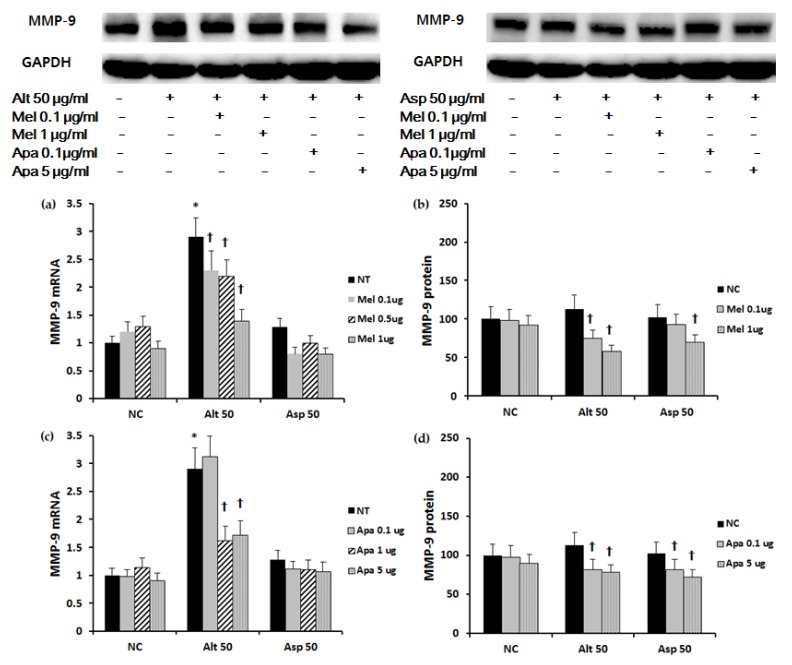
Effect of melittin and apamin on the expression of MMP-9 mRNA and protein in nasal fibroblasts. *Alternaria* induced MMP-9 mRNA expression was significantly inhibited by (**a**) melittin and (**c**) apamin. (**b**,**d**) Melittin and apamin tended to inhibit the production of MMP-9 protein. Alt 50: *Alternaria* 50 μg/mL; Asp 50: *Aspergillus* 50 μg/mL; NC: negative control; NT: non-treated; Mel: melittin; Apa: apamin; μg: μg/mL; * *p* < 0.05 compared with negative control; † *p* < 0.05 compared with the non-treated group.

Collagen type I mRNA and protein expression was significantly increased with 50 μg/mL of *Aspergillus. Aspergillus* induced collagen type I mRNA and protein expression was significantly suppressed when treated with melittin. Apamin suppressed both *Alternaria* and *Aspergillus* induced collagen type I mRNA and protein expression (Figure 3). When the nasal polyp fibroblasts were stimulated with *Alternaria* TIMP-1 mRNA and protein expressions were significantly increased. However, TIMP-1 mRNA and protein expressions were not significantly increased by stimulation with *Aspergillus*. TIMP-1 mRNA expression induced by *Alternaria* was significantly inhibited by apamin in a dose dependent manner, and TIMP-1 protein expression was also significantly inhibited by melittin and apamin in a dose dependent manner (Figure 4). *Alternaria* induced MMP-9 mRNA expression, but *Aspergillus* did not induce MMP-9 mRNA expression. *Alternaria* induced MMP-9 mRNA expression was significantly inhibited by meittin and apamin. Although fungi did not influence the production of MMP-9 protein, melittin and apamin tended to inhibit the production of MMP-9 from nasal polyp fibroblasts (Figure 5).

### 2.4. Effect of Melittin and Apamin on Phosphorylation of Smad 2/3 and p38 MAPK

**Figure 6:**
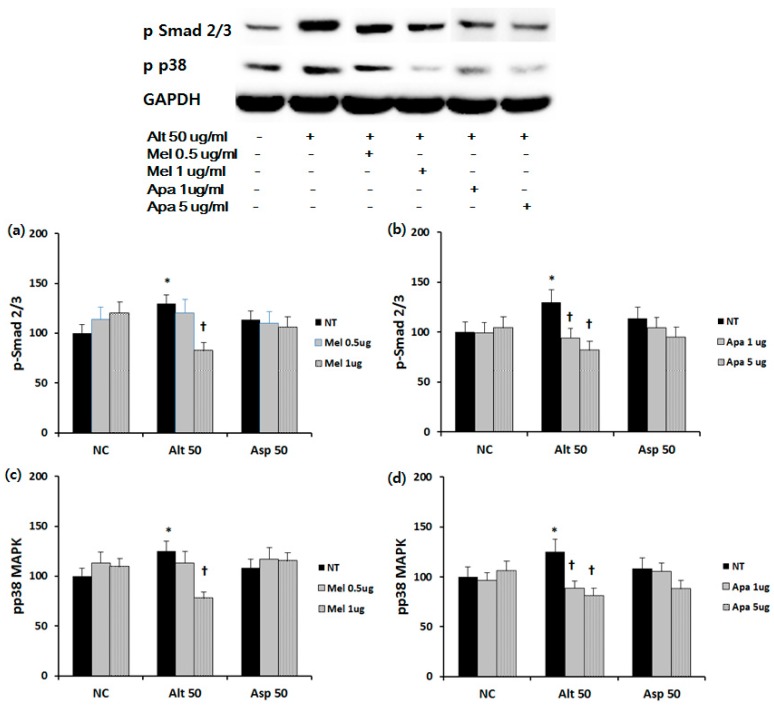
Effect of melittin and apamin on phosphorylation of Smad 2/3 and p38 MAPK expression in nasal fibroblasts. Phosphorylation of Smad 2/3 and p38 MAPK was measured using Western blotting and density analysis. *Alternaria* induced Smad 2/3 and p38 MAPK phosphorylation was significantly inhibited by (**a**,**c**) melittin and (**b**,**d**) apamin. Alt 50: *Alternaria* 50 μg/mL; Asp 50: *Aspergillus* 50 μg/mL; NC: negative control; NT: non-treated; Mel: melittin; Apa: apamin; μg: μg/mL; * *p* < 0.05 compared with negative control; † *p* < 0.05 compared with the non-treated group.

To determine the inhibitory mechanism of ECM production, we identified the effect of melittin and apamin on phosphorylation of Smad2/3 and p38 MAPK. Duration of treatment was 30 min for p-p38 and 60 min for pSmad 2/3. The densitometric quantification results showed that *Alternaria* potently induced the activation of Smad 2/3 and p38 MAPK. When the fibroblasts were treated with 1 μg/mL of melittin, Smad 2/3 (approximately 30.9%), and p38 MAPK (approximately 37.4%) expressions were significantly suppressed. Apamin also significantly suppressed Smad 2/3 (approximately 27.4% and 36/4% at 1 and 5 μg/mL of apamin) and p38 MAPK (approximately 29.2% and 34.7% at 1 and 5 μg/mL of apamin) expressions (Figure 6).

## 3. Discussion

BV has been used as a traditional medicine with satisfactory results for the treatment of some inflammatory, cancer, and immune related diseases [6,7,12]. Melittin and apamin are the most well-known components of BV. Melittin is the main component of BV with hyaluronidase and phospholipase A2 [13]. Melittin is the active component of apitoxin with antimicrobial, anti-inflammatory, and anti-atherosclerotic properties [8,9]. Apamin is a peptide neurotoxin with a crucial role in repetitive activities in neurons, inducing alpha-adrenergic, cholinergic, purinergic, and neurotensin-induced relaxation [7,14]. In this study, we tried to determine the effect of melittin and apamin on fungi induced chemical mediator and extraceullar matrix production from nasal fibroblasts. 

Fungi have been associated with upper and lower airway inflammatory diseases and *Alternaria* and *Aspergillus* are commonly found in the nasal secretion and the respiratory tract [15]. Fungi can induce chemical mediator production through the interaction with toll-like receptors (TLRs) [3]. IL-6 and IL-8 productions were increased with *Alternaria* and production of these chemical mediators was significantly inhibited when the fibroblasts were treated with apamin. BV also significantly inhibited the production of IL-6 and IL-8. BV encompasses a mixture of many types of compounds, proteins, peptides, and enzymes. However, BV did not strongly inhibit chemical mediator production compared to apamin in nasal fibroblasts. IL-6 production was more strongly inhibited by BV (50.1% at 1 μg/mL of BV vs. 42.6% at 1 μg/mL of apamin), and IL-8 production was more strongly inhibited by apamin (28.8% at 1 μg/mL of BV vs. 38.7% at 1 μg/mL of apamin). Although we cannot explain the exact cause of this discrepancy, some components of BV may enhance the anti-inflammtory effect and the other components may suppress the anti-inflammatory effect of apamin. Anti-inflammatory properties of BV for treating skin disease, neurodegenerative disease, and joint diseases have been commonly studied, and these anti-inflammatory effects are associated with decreased expression of TLRs, chemical mediators, nitric oxides, and phosphorylation of inflammatory transcription factors [7]. According to the previous study, *Alternaria* induces the production of chemical mediators through TLR 2 and TLR5 [16]. Although we do not know the action mechanism of apamin and melittin, they may not influence the expression of TLR2 or 5 or the concentration of melittin used in this study may not enough to suppress the production of chemical mediators from nasal fibroblasts. 

The damage to mucosal epithelium, ECM accumulation, and inflammatory cell infiltration are important pathologic findings of nasal polyps [2]. Fibroblasts are the cellular source of ECM and they are involved in the development of nasal polyps. The MMP-9 level was elevated in nasal polyps and the TIMP-1 level was elevated in chronic rhinosinusitis [17]. We performed a kinetic study with *Alternaria* and *Aspergillus* for 8, 24, and 48 h. Collagen type I, TIMP-1, and MMP-9 mRNA expression levels were the highest at 24 h stimulation. Expression levels of these ECM proteins were highest at 6 h after stimulation with fungi. However, fungi did not influence the fibronectin mRNA and protein expression in nasal fibroblasts [18]. Therefore, we evaluated collagen type I, TIMP-1, and MMP-9 mRNA expression in nasal fibroblasts at 24 h and their protein expression at 6 h after stimulation with fungi. In this study, the expression pattern of ECM was different depending on the type of fungi used for stimulation. These differences may be associated with the unique molecular pattern, the peptides, or the immune triggering components of fungi [19]. Also, different fungi may interact with different pattern recognition receptors, such as TLRs, protease activated receptors, or G-protein-coupled receptors. TLR2, TLR4, and TLR9 are the main TLRs involved in sensing the fungal components [20]. *Alternaria* and *Aspergillus* enhance the production of chemical mediators through TLR4 in nasal epithelial cells [21]. Kao et al. suggested that TLR4 triggering activates the MAPK signaling pathway, which cross-talks with the Smad2 cascade and promotes the production of ECM [22]. When the nasal fibroblasts were stimulated with fungi, collagens type I, TIMP-1, and MMP-9 mRNA and/or protein expressions were increased. Also, this study showed that *Alternaria* can induce Smad 2/3 hyperphosphorylation in a TGF-β independent manner and p38 MAPK hyperphosphorylation. Our results suggest that *Alternaria* can directly activate the Smad 2/3 cascade or indirectly induce phosphorylation of Smad 2/3 through the TLR4/MAPK signaling pathway. 

Melittin and apamin have been known to inhibit ECM production and tissue fibrosis from kidney and liver [10,11,23]. In this study, the inhibition of ECM expression in nasal fibroblasts by melittin and apamin differed depending on the types of ECM mRNA and protein. Although melittin and apamin show anti-fibrotic properties, they have different pharmacological characteristics, chemical structures, and they may control different signaling pathways. Melittin and apamin showed different patterns in suppressing the pro-fibrotic activity in TGF-β treated fibroblasts. Melittin attenuates fibrogenesis by inhibiting NF-κB and AP-1 dependent collagen type I and MMP-9 expression [10,24]. Apamin attenuates fibrogenesis by inhibiting phosphorylated Smad 2/3 and Smad dependent ECM deposition [25]. Melittin and apamin inhibited *Alternaria* induced phosphorylation of Smad 2/3 and MAPK in nasal fibroblasts. Although we do not know whether melittin and apamin directly suppress phosphorylation of Smad 2/3, melittin and apamin can directly or indirectly inhibit the *Alternaria* induced Smad 2/3 cascade. 

The principal finding of this study is the anti-inflammatory and anti-fibrotic effects of melittin and apamin. Fungi can induce production of chemical mediators and ECM deposition in nasal fibroblasts. The production of these chemical mediators and ECM production were inhibited by melittin and apamin. In particular, melittin and apamin inhibited ECM production through direct suppression of the Smad cascade or indirect inhibition of Smad 2/3 phosphorylation through the MAPK signaling pathway. These results suggest a novel pharmacological rationale for the treatment of fungi induced airway inflammatory diseases. Because BV contains melittin and apamin, BV may have strong anti-inflammatory and anti-fibrotic effects, and could be a good candidate as a therapeutic agent for airway inflammatory diseases.

## 4. Materials and Methods

### 4.1. Isolation of Primary Nasal Polyp Fibroblasts

Primary nasal fibroblasts were isolated from 11 patients (7 men and 4 women; 43.5 ± 8.2 years) with chronic rhinosinusitis with nasal polyps during endoscopic sinus surgery. Subjects were excluded if they had an active inflammation, allergy, or aspirin hypersensitivity, had received antibiotics, antihistamine, steroids, or other medications for at least four weeks preceding the surgery. Allergy status was defined using the skin prick test. The study was approved by the Institutional Review Board of Daegu Catholic University Medical Center. A duly completed written informed consent form that outlined the objectives of the research and experiments was obtained from each patient. 

The tissues were cut into 0.3 to 0.5 mm fragments and washed with phosphate buffered saline. These tissues were suspended and cultured in Dulbecco’s Modified Eagle’s Medium F-12 (DMEM/F-12) (Gibco, Grand Island, NY, USA) that contained 10% fetal bovine serum, penicillin at 100 U/mL, streptomycin at 100 μg/mL, and amphotericin B at 1.5 μg/mL at 37 °C and 5% CO_2_. The second to third passages of fibroblasts were used for this experiment.

### 4.2. The Cytotoxic Effect of BV, Melittin, and Apamin on Nasal Polyp Fibroblasts

The cytotoxic effect of BV (melittin comprise approximately 50% and apamin comprise 3% of dried BV) (Chung Jin Biotech Co., Ansan, Korea) [26], melittin (Enzo Life Sciences AG, Lausen, Switzerland), and apamin (Sigma-Aldrich, St. Louis, Mo, USA) was evaluated using a CellTiter-96^®^ aqueous cell proliferation assay kit (Promega, Madison, WI, USA). On a 96-well microstate plate, NP fibroblasts were cultured in the presence of 0.1, 1, 3, and 5 μg/mL of BV, 0.1, 1, 3, and 5 μg/mL of melittin, and 0.1, 1, 5, and 10 μg/mL of apamin for 24 h at 37 °C in a 5% CO_2._ The reduced tetrazolium compound produces a colored formazan product due to the mitochondrial activity in the cell. The amount of formazan is directly proportional to the number of viable cells. Color intensities were assessed with a fluorescence microplate reader at 490 nm.

### 4.3. The Effect of Bee Venom, Melittin, and Apamin on IL-6 and IL-8 Production from Nasal Polyp Fibroblasts 

The fibroblasts were incubated with endotoxin removed *Alternaria alternate* and *Aspergillus fumigatus* at 50 and 25 μg/mL, respectively (Greer Lab, Lenoir, NC, USA). After 24 h of stimulation, the cell culture supernatants and cells were harvested and stored at −70 °C until they were assayed. To determine the effect of melittin and apamin on the production of Interleukin (IL)-6 and IL-8, fibroblasts were incubated with or without various concentrations of melittin and apamin. IL-6 and IL-8 were quantified by using commercially available ELISA kits (R&D system, Minneapolis, MN, USA). 

### 4.4. Real Time Reverse Transcription–Polymerase Chain Reaction (RT-PCR) for ECM mRNA from Nasal Polyp Fibroblasts

Fibroblasts were exposed to fungi with or without melittin and apamin for 24 h. The total RNA was extracted from fibroblasts with Trizol reagent (Invitrogen, Carlsbad, CA, USA) according to the manufacturer’s instructions. Total RNA, 1 μg, was reverse transcribed using SuPrimeScript RT Premix (Genetbio Inc., Daejeon, Korea) and Quantitative PCR was then carried out on a mini opticon system (Bio-Rad Lab., Hercules, CA, USA) according to the manufacturer’s protocol. The forward and reverse primers were as follows: β-actin, 5-ACAGGAAGTCCCTTGCCATC-3 and 5-AGGGAGACCAAAAGCCTTCA-3; α-SMA, 5-ATAGAACATGGCATCATCACCAAC-3, and 5-GGGCAACACGAAGCTCATTGTA-3; fibronectin, 5-GCCAGATGATGAGCTGCAC-3, and 5-GAGCAAATGGCACCGAGATA-3; tissue inhibitors of matrix metalloproteinase-1 (TIMP-1) 5-CCTTATACCAGCGTTATGAGATCAA-3 and 5-AGTGATGTGCAAGAGTCCATCC-3; and matrix metalloproteinase-9 (MMP-9), 5-ATTTCTGCCAGGACCGCTTCTACT-3, and 5-CAGTTTGTATCCGGCAAACTGGCT-3. The cDNA was amplified with initial denaturation at 95 °C for 10 min, followed PCR for 40 cycles of 95 °C for 5 s, 58 °C for 30 s, and finally one cycle of melting curve following cooling at 60 °C for 60 s. To confirm the amplification specificity, the PCR products from each primer pair were subjected to a melting curve analysis. Analysis of relative gene expression was performed by evaluating q-RT-PCR data by the 2(-DDCt) method. The gene expression levels were determined by normalization relative to β-actin expression.

### 4.5. Western Blot Analysis of Nasal Polyp Fibroblasts 

Nasal fibroblast lysates were subjected to sodium dodecyl sulfate polyacrylamide gel electrophoresis and transferred onto NC membranes (Bio-Rad, Berkeley, CA, USA). Membranes were blocked with 5% skim milk solution and they were incubated with antibodies against MMP-9 (Cell signaling, Beverly, MA, USA), collagen type I, fibronectin, TIMP-1, phosphorylated Smad (pSmad) 2/3, p38 mitogen-activated protein kinase (MAPK), and GAPDH (Santa Cruz Biotechnology, Santa Cruz, CA, USA). After incubation for 1 h, the membranes were washed and then treated with peroxidase-conjugated anti-rabbit immunoglobulin G (Santa Cruz Biotechnology). Bands were visualized using horseradish peroxidase conjugated secondary antibodies and an ECL system (Pierce, Rockford, IL, USA). The band densities were measured using the multi Gauge v.2.02 software (Fujifilm, Tokyo, Japan). The band intensities were expressed as a percentage of treated cells versus untreated cells.

### 4.6. Statistical Analysis

The experimental data are presented as mean ± SE. The statistical significance of the differences between control and experimental data was analyzed using paired or unpaired Student’s *t*-test and one-way analysis of variance followed by Tukey’s test (SPSS ver. 21.0, SPSS Inc., Chicago, IL, USA). *p* value < 0.05 was considered to indicate a statistically significant difference. All results were obtained from at least four independent individuals and every experiment was performed in duplication.
